# Development of a mouse embryonic stem cell model for investigating the functions of the linker histone H1‐4

**DOI:** 10.1002/2211-5463.13750

**Published:** 2024-01-11

**Authors:** Abed Alkarem Abu Alhaija, Imtiaz Nisar Lone, Esin Ozkuru Sekeroglu, Tugce Batur, Dimitar Angelov, Stefan Dimitrov, Ali Hamiche, Elif Nur Firat Karalar, Muhammed Erdem Ercan, Tamer Yagci, Hani Alotaibi, Muhammed Kasim Diril

**Affiliations:** ^1^ Department of Molecular Biology and Genetics, Faculty of Basic Sciences Gebze Technical University Turkey; ^2^ Izmir Biomedicine and Genome Center Turkey; ^3^ Izmir International Biomedicine and Genome Institute Dokuz Eylül University Izmir Turkey; ^4^ Laboratoire de Biologie et de Modélisation de la Cellule LBMC, CNRS UMR 5239 Université de Lyon, Ecole Normale Supérieure de Lyon France; ^5^ Roumen Tsanev Institute of Molecular Biology Bulgarian Academy of Sciences Sofia Bulgaria; ^6^ Institute for Advanced Biosciences, Inserm U1209, CNRS UMR 5309 Université Grenoble Alpes France; ^7^ Institut de Génétique et Biologie Moléculaire et Cellulaire (IGBMC) UdS, CNRS, INSERM Strasbourg France; ^8^ Department of Molecular Biology and Genetics Koç University Istanbul Turkey; ^9^ Department of Medical Biology, Faculty of Medicine Dokuz Eylül University Izmir Turkey

**Keywords:** cellular model, CRISPR/Cas9, H1.4, linker histones, mES cells, Rahman syndrome

## Abstract

The linker histone H1 C‐terminal domain (CTD) plays a pivotal role in chromatin condensation. *De novo* frameshift mutations within the CTD coding region of H1.4 have recently been reported to be associated with Rahman syndrome, a neurological disease that causes intellectual disability and overgrowth. To investigate the mechanisms and pathogenesis of Rahman syndrome, we developed a cellular model using murine embryonic stem cells (mESCs) and CRISPR/Cas9 genome engineering. Our engineered mES cells facilitate detailed investigations, such as H1‐4 dynamics, immunoprecipitation, and nuclear localization; in addition, we tagged the mutant H1‐4 with a photoactivatable GFP (PA‐GFP) and an HA tag to facilitate pulldown assays. We anticipate that these engineered cells could also be used for the development of a mouse model to study the *in vivo* role of the H1‐4 protein.

## Graphical abstract

To study Rahman syndrome, we developed a cellular model by genetically engineering mES cells using CRISPR. The modified cells express human H1.4 variants labeled with PA‐GFP and HA tags that allow protein dynamics measurements and can be used in biochemical studies. The mES cells can also be differentiated into various lineages or used in the generation of mouse models for Rahman syndrome.

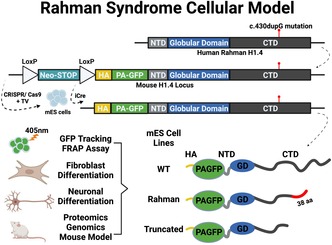

**Abbreviations**
Cas: CRISPR‐associated protein
CDS: coding sequence
CRISPR: clustered regularly interspaced short palindromic repeats
CTD: C‐terminal domain
GD: globular domain
GSK3: glycogen synthase kinase 3
H1.4: H1.4 linker histone, cluster member
H1‐4: H1‐4 linker histone protein
LIF: leukemia inhibitory factor
LSL: lox‐stop‐lox
MEK: mitogen‐activated protein kinase
mESC: mouse embryonic stem cells
NTD: N‐terminal domain
PA‐GFP: photoactivatable green fluorescent protein
PFU: plaque‐forming unit
RA: retinoic acid
WT: wild‐type

Organisms face the fundamental challenge of compacting lengthy DNA molecules, such as the 2‐m‐long human DNA, into the confined space of a cell nucleus in an organized and structured manner. This intricate process relies on DNA's interactions with histones and subsequent chromatin assembly. In this context, crucial DNA‐templated functions such as gene expression, DNA repair, replication, and mitosis occur. Biological events in the nucleus, such as transcription and replication, require unfolding and a subsequent refolding of the chromatin. Maintaining nuclear homeostasis requires an intricate and adaptable chromatin organization. Thus, understanding the complexity of chromatin organization and plasticity is essential for gaining profound insights into DNA functionality and deciphering how disruptions in DNA‐templated processes contribute to human diseases.

The primary protein components of chromatin in all eukaryotic cells are the basic proteins termed histones [1]. The core histones form an octamer (two of each H2A, H2B, H3, and H4) around which DNA is wrapped to form nucleosomes. In contrast, the linker histones primarily interact with the linker DNA that connects nearby nucleosomes [2]. The linker histone is a fundamental player in chromatin compaction and dynamics. Mammals exhibit a remarkable diversity of linker histones, with 11 distinct isoforms, making them the most diverse group among histones [3]. Structurally unrelated to the core histones, linker histones are lysine‐rich proteins and one of the most positively charged proteins in cells [4]. They have a tripartite structure composed of a less conserved short unstructured N‐terminal domain (NTD) of around 20–35 amino acids, a highly conserved, stably folded central globular domain (GD) of approximately 80 amino acids, and a less conserved, long, and highly disordered C‐terminal domain (CTD) of approximately 100 amino acids [5, 6, 7]. The CTD amino acid compositions are similar across species and subtypes and mainly consist of positively charged lysines, accounting for a net positive charge of 30–50 [3]. The CTD exhibits a random coil structure in an aqueous environment; however, it can adopt a folded structure upon interacting with DNA or in the presence of secondary structure stabilizers [8]. It is essential for high‐affinity nucleosome binding and chromatin condensation [9, 10, 11].

Lately, the crystal and cryo‐EM structures of a 197‐bp nucleosome in complex with vertebrate linker histone H1 were solved. In addition, recent data from biochemical experiments revealed that histone H1 shifts the 3D organization of the nucleosome by drawing the two linkers together and reducing their flexibility [12, 13, 14, 15]. The H1 CTD interacts mainly with a single linker, while the H1 GD contacts the nucleosome dyad and both linkers, associating more closely with the CTD‐distal linker. The nucleosome dyad is very likely to determine the CTD collapse on distinct DNA arms [16]. These findings reveal that H1 imposes a remarkable degree of asymmetry on the nucleosome, influencing the assembly and architecture of higher‐order structures [17].

Recently, *de novo* occurring frameshift mutations within the region coding for the CTD of the H1.4 linker histone (also known as H1E, HIST1H1E) were identified and associated with Rahman syndrome, a neurodevelopmental disorder (OMIM #617537). Rahman syndrome is characterized by intellectual disability and diverse clinical anomalies and follows an autosomal dominant pattern [18]. Rahman mutations, including small insertions, deletions, or duplications, tend to cluster within a 94‐bp region in the CTD of the gene [18]. Consequently, the mutated H1‐4 protein becomes truncated and has a reduced net positive charge, featuring a stretch of 38 amino acids in the C‐terminus of the tail that is present in all affected subjects [18]. Fibroblasts derived from affected individuals manifested distinct abnormalities, including enhanced chromatin relaxation, reduced methylation of specific lysine residues on histone H3 (H3K4me2, H3K9me3, H3K27me3), and decreased binding of HP1β protein [19]. These alterations strongly suggest impaired heterochromatin formation and aberrant chromatin dynamics in Rahman syndrome [19]. These discoveries shed light on the underlying molecular mechanisms contributing to the pathogenesis of Rahman syndrome and provide a foundation for further investigation into potential therapeutic strategies.

Understanding the role of linker histones and their CTD, both in normal development and in the context of Rahman syndrome, is essential for unraveling the mechanisms of genome folding and the molecular basis of the disease. However, no cellular model is available except for the fibroblasts from Rahman syndrome patients. The availability of such a cellular model would help to gain insights into the functional consequences and molecular pathways associated with the H1.4 mutation, thus providing valuable information for future therapeutic strategies and interventions.

In this article, we describe the generation of a cellular model for Rahman syndrome that we developed using murine embryonic stem cells (mESCs) and CRISPR/Cas9 genome engineering technology. This model allows for conditional expression of the mutant H1‐4 protein under its endogenous promoter, mimicking physiological conditions. The engineered cells express the mutated H1‐4, labeled with a PA‐GFP and an HA tag. This labeling would enable the study of the H1‐4 exchange rate and facilitate immunoprecipitation experiments. Since no specific antibodies are available for the linker Histone H1‐4, the tagging of the Rahman mutant allows us to detect it by immunofluorescence and by western blotting specifically. These tags could also be used for the pulldown assays that will help identify the proteins interacting with the Rahman mutant H1‐4. Using this cellular model will make it feasible to study the impact of the H1.4 mutation on chromatin organization, cellular proliferation, and nuclear stability. Moreover, the engineered mES cells provide a unique platform for establishing a mouse model that would enable comprehensive investigations into the diverse tissue‐specific expression of these mutant variants of H1‐4.

## Materials and methods

### mESC culture

B6/BLU mESCs (American Type Culture Collection, Manassas, VA, USA) were cultured on a gelatinized (0.2% gelatin in PBS) culture plate in complete 2i medium. The medium was prepared by mixing 1× DMEM/F12, 1× neurobasal medium, 1% fetal bovine serum, 0.5× B27 supplement, 0.5× N2 supplement, 7.5% bovine serum albumin (BSA), 1× penicillin–streptomycin, 55 μm β‐mercaptoethanol, 3 μm GSK3 inhibitor (CHIR99021), 1 μm MEK inhibitor (PD0325901), and 40 ng·mL^−1^ of LIF (Lab made). The cells were maintained at 37 °C with 5% CO_2_, and the growth medium was refreshed daily while passaging was performed every 2–3 days [20].

### Targeting strategy and target vector design

**Fig. 1:**
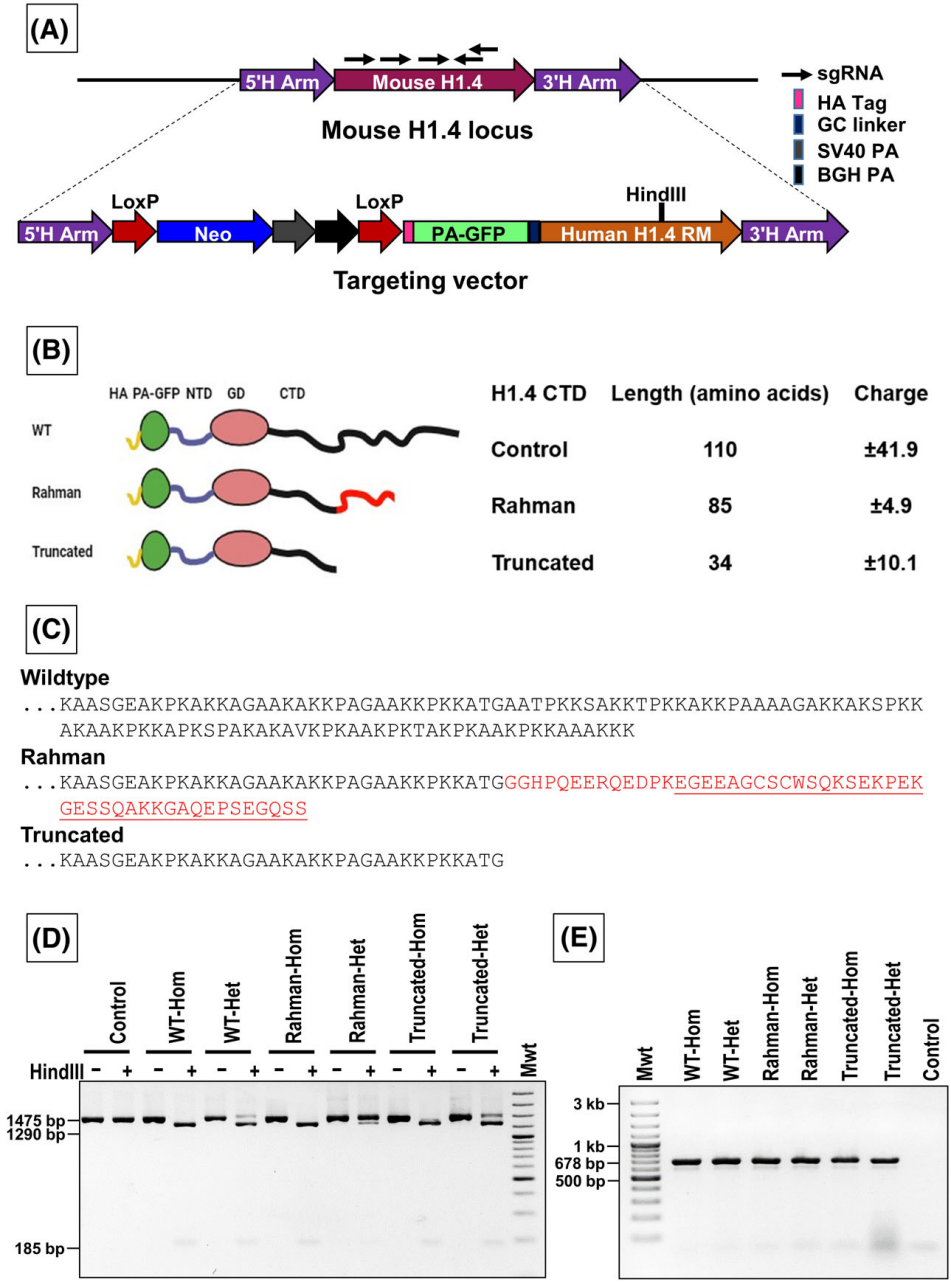
H1.4 targeting strategy and genotyping. (A) Illustrative diagram depicting the H1.4 targeting strategy: Five guide RNAs (sgRNAs) were used to target the mouse H1.4 gene. The targeting vector carries a cassette containing the neomycin resistance gene for selection and PA‐GFP‐H1.4. The Rahman mutation is incorporated in H1.4, and the whole cassette is flanked by 5′ and 3′ homology arms (H Arm). The neomycin gene is placed under the control of the endogenous H1.4 promoter and carries a stop codon. The expression of the transgene PA‐GFP‐H1.4 is induced by Cre recombination, which removes the neomycin cassette and places PA‐GFP‐H1.4 under the control of the endogenous promoter. (B) Pictorial representation of different H1‐4 fusion protein variants (on the left). The length and net charge of the C‐terminal domain in different mutated H1‐4 protein variants compared to the wild‐type protein (on the right). The net charge was calculated using the tool available at https://www.protpi.ch/. (C) Amino acid sequence of the C‐terminal domain of the H1‐4 histone protein variants. The Rahman mutation site and sequence are highlighted in red. (D) HindIII (introduced by silent mutation) digestion of the 3′ end screening PCRs: 3′ end screening PCR has reverse primer outside the targeted locus and forward primer in the H1.4 gene; positive results of this PCR confirm that the transgene is inserted at the correct locus at the 3′ end. All depicted clones exhibit successful incorporation of PA‐GFP‐H1.4 at the correct location of the mouse gene. Since HindIII is present in the transgene only, digestion of the 3′ end PCR with HindIII allows us to confirm the zygosity for the transgene. The heterozygous clone exhibits the release of three fragments, while the homozygous clone shows the release of only two fragments. (E) Agarose gel electrophoresis of the 5′ end screening PCR: For 5′ PCR, one primer is located outside the targeted locus and one within the antibiotic resistance gene. Positive results of this PCR confirm that the transgene is inserted at the correct locus at this end.

The pACAGW‐H2B‐PA‐GFP‐AAV vector was generously provided by S. Huet. The 5′ and 3′ H1.4 mouse homology arms and the human H1.4 gene tagged with an HA tag were commercially synthesized (Eurofins, Bağcılar/İstanbul, Turkish) and cloned into the pUC57 backbone. In addition, the synthesized vector includes a promoterless neomycin gene flanked by two loxP sites inserted before the H1.4 coding sequence (CDS). The PA‐GFP gene was amplified via PCR from the pACAGW‐H2B‐PA‐GFP‐AAV vector and introduced at the 5′ of the H1.4 gene utilizing SpeI and AgeI restriction enzymes. Furthermore, a GC linker was introduced between the PA‐GFP and the human H1.4 gene through site‐directed mutagenesis (Fig. 1A and Fig. S1). H1.4 sgRNAs (Table 1) were designed by the Chopchop webtool (https://chopchop.cbu.uib.no/) and cloned into pX458 expressing Cas9 nuclease fused to green fluorescent protein (GFP; Addgene #48138, Watertown, MA, USA) according to the protocol outlined in Ran *et al*. [21]. To create our cellular model, we selected the Rahman mutation with the highest incidence (c.430dupG duplication/deletion) and introduced it into mES cells. In addition to the Rahman mutant, we also created a truncated mutant in which a stop codon is introduced at the site where the most prevalent Rahman mutation takes place.

**Table 1:** H1.4 sgRNA target sequences. sgRNA target sequences used for targeting the mouse endogenous locus.

sgRNA	Sequence (5′–3′)
1	**CACCG** CTTGACGGGTGTCTTCTCGG
2	**CACCG** TTTGCGCTTCGCGCCACCTG
3	**CACC** GGTGTCCGAACTCATCACCA
4	**CACCG** CTTCTCCACATCGTACCCCG
5	**CACCG** TTACCGTTTTCGCCTTGGCA

The bold letters represent overhangs for ligation.

### Genotyping

DNA extraction from the collected cells of the selected clones was performed using a lysis buffer composed of TE buffer, 2% Tween 20, and 1 mg·mL^−1^ proteinase K. The cells were lysed for 1 h at 55 °C. Subsequently, heat inactivation was carried out at 95 °C for 15 min [22]. Confirmation of successful recombination at the mouse H1.4 locus involved conducting two PCR reactions using specific primers (Table 2) that separately tested the recombination at the 5′ and 3′ homology arms. The positive colonies that exhibited both PCR products were selected for the subsequent genotyping steps. The PCR products of the 3′ homology arm were digested using the HindIII restriction enzyme to determine the H1.4 zygosity. Through this analysis, homozygous and heterozygous clones were identified and selected for further experimentation.

**Table 2:** Oligos used for the cloning and genotyping analysis. This table presents a list of oligos utilized throughout the study.

PCR product	Primers	Oligo 5′–3′ sequence
PA‐GFP	PA‐GFP‐F	AGGACTAGTATGGTGAGCAAGGGCGAG
	PA‐GFP‐R	AGGACCGGTCTTGTACAGCTC GTCCATGCC
H1.4 3′ homology arm	3′ Homology‐F	CGCTCCTTGTCCTTCTGTTTGTT
	3′ Homology‐R	CGTCAAGAAGAAGGCCCGCAA
H1.4 5′ homology arm	5′ Homology‐F	GCTCAATAGGCAGGACTCTCG
	5′ Homology‐R	GTGCCCAGTCATAGCCGAATA
Oct4 RT‐qPCR	Oct4‐F	TCTTTCCCCAGGCCCCCGGCTC
	Oct4‐R	TGCGGGCGGACATGGGGAGATCC
Sox2 RT‐qPCR	Sox2‐F	CGCGGCGGAAAACCAAGACG
	Sox2‐R	GCCGGCGCCCACCCCAACC
Mouse H1.4 RT‐PCR	mH1.4‐F	GCCAAGGCGAAAACGGTAAA
	mH1.4‐R	TGCGGTTTTCTTTGGCTTAGC
GAPDH RT‐qPCR	GAPDH‐F	TTCACCACCATGGAGAAGGC
	GAPDH‐R	GGCATGGACTGTGGTCATGA
PA‐GFP‐H1.4 RT‐PCR	PA‐GFP‐H1.4‐F	AGGACTAGTATGGTGAGCAAGGGCGAG
	PA‐GFP‐H1.4‐R	TTGTTGGGCTTCTAAGCAGTTGG

### Cell transfection, clone selection, and Cre recombinase adenovirus infection

The B6/BLU mES cells were employed to generate conditional H1.4 knockout cells. In brief, the cells were transfected with a plasmid cocktail containing the sgRNA vectors and the H1.4 targeting vector using the Xfect™ mESC Transfection Reagent. After 48 h of transfection, the cells were subjected to selection using Geneticin (200 μg·mL^−1^) for 2 weeks. Drug‐resistant colonies exhibiting an undifferentiated morphology were carefully picked, expanded, and subjected to appropriate genotyping procedures. To achieve inducible expression of the human histone H1.4 gene, cells derived from the selected clones were amplified in complete 2i media on gelatinized six‐well plates. Subsequently, the cells were treated with Cre Recombinase Adenovirus (1 × 10^10^ PFU·mL^−1^) (Cat. No. 1045; Vector Biolabs, Malvern, PA, USA). After 24 h of adeno‐Cre treatment, cells were washed with PBS, and fresh media was added to the culture. The cells were incubated for another 36 h to allow the expression of the transgenes before harvesting them for analysis using RT‐PCR and western blotting.

### Growth curve analysis

The potential effects of H1.4 knockout or the expression of different H1‐4 mutants on mES cell proliferation were assessed by comparing the growth rates of the mutants and their wild‐type counterparts. The experiment was repeated three times to ensure the robustness and reliability of the results. About 50 000 cells were seeded on a gelatinized 12‐well plate in complete 2i medium, and their proliferation was monitored for 4 days. Daily cell collections were performed, and cell counts were obtained. Finally, based on the collected data from each repeat, a growth curve was generated to plot the cell growth during the experiment.

### RT‐PCR

Total RNA was extracted from the generated clones using an RNA purification kit (Macherey‐Nagel, Düren, Germany). The isolated RNA was then used as a template for cDNA synthesis, employing the cDNA Synthesis Kit from (Thermo Fisher Scientific, Waltham, MA, USA). The resulting cDNA was used for PCR amplification to evaluate the expression of the PA‐GFP‐H1.4 fusion gene and the mouse H1.4 (Table 2). The PCR products were subsequently examined on an agarose gel. Additionally, the expression levels of pluripotency markers (Sox2 and Oct4) (Table 2) were assessed using quantitative qPCR, employing the RealQ Plus 2× Master Mix Green Without ROX from Amplicon (Odense M, Denmark). The experiment was repeated three times to ensure the robustness and reliability of the results.

### Western blot

Cell extracts were prepared using cells derived from the created clones. The cells were lysed in RIPA buffer (25 mm Tris–HCl pH 7.6, 150 mm NaCl, 5 mm EDTA, 1% NP40 or 1% Triton X‐100, 1% sodium deoxycholate, 0.1% SDS, 1 mm DTT, 1 mm phenylmethylsulfonyl fluoride, and 1× protease inhibitors) for 30 min on ice. The lysates were loaded onto SDS gel and subsequently transferred onto an immobilon‐P membrane (Millipore Sigma, Burlington, MA, USA). The membrane was then blocked with 5% milk and probed with a mouse anti‐GFP antibody (Cat. No. 1181446000; Roche, Basel, Switzerland) to detect the PA‐GFP‐H1‐4 fusion protein. The mouse anti‐tubulin antibody (Cat. No. Ab56676; Abcam, Cambridge, UK) was used for internal control. Subsequently, the membranes were incubated with goat anti‐mouse HRP (Cat. No. Ab205719; Abcam), and then the Clarity Western ECL Substrate kit (Bio‐Rad, Hercules. CA, USA) was used for protein detection. Blots were imaged using the ChemiDoc Imaging System from Bio‐Rad.

### Immunofluorescence

The cells derived from the created clones were cultured on coverslips coated with 0.01% poly‐l‐lysine or 0.2% gelatin. The cells were fixed with 3% paraformaldehyde for 10 min, permeabilized with 0.5% Triton X‐100, and then blocked in a blocking buffer (1% BSA, 22.52 mg·mL^−1^ glycine in PBST, and 0.1% Tween 20 in PBS buffer) for 30 min. Next, the cells were incubated with the primary antibody for 1 h at room temperature. After three washes with PBST, the cells were incubated with an Alexa Fluor secondary antibody for 1 h at room temperature. The coverslips were counterstained with DAPI and mounted on slides. Finally, images were captured using either fluorescent or confocal microscopy.

### Cell cycle analysis

The cultured clones in a gelatinized six‐well plate were detached using trypsin and collected by centrifugation. Cell pellets were washed in PBS and resuspended in ice‐cold 100% ethanol. The cells were incubated overnight at −20 °C and then washed three times with PBS. Finally, the pellets were dissolved in a staining solution containing 50 μg·mL^−1^ propidium iodide (PI) (Thermo Fisher Scientific), 1 mg·mL^−1^ RNAase A, and 0.05% Triton X. PI binding was assessed in the Per‐CP Cy5.5 channel using flow cytometry. Cell cycle analysis was performed using flowjo v10 software (FlowJo, Ashland, OH, USA) with the Watson Pragmatic algorithm. The experiment was repeated three times to ensure the robustness and reliability of the results.

### Differentiation of mES cells to fibroblast

The created clones were differentiated into fibroblasts using a protocol adapted from D'Angelo *et al*. [23]. In this protocol, mES cells were initially cultured on a gelatinized 10 cm plate at a low density of 30–40% confluence in DMEM media supplemented with 10% FBS. Simultaneously, the cells were treated with 1 μm retinoic acid (RA) for 10 days. The medium was refreshed every 2 days during this period. After 10 days, the cells formed a compact monolayer, which was trypsinized and disaggregated. The resulting single‐cell suspension was then reseeded in an uncoated 10‐cm plate, allowing the fibroblasts to quickly attach within approximately 1 h. The medium was changed twice to eliminate floating cells in the medium. These fibroblast cells were further propagated in DMEM supplemented with 10% FBS and analyzed by immunofluorescence using an anti‐HA antibody (Cat. No. 34502100; Roche) and Alexa Fluor™ 488 phalloidin (Cat. No. A12379; Invitrogen, Waltham, MA, USA) for F‐actin labeling.

### PA‐GFP photoactivation

For PA‐GFP photoactivation, cells expressing the PA‐GFP‐H1.4 fusion protein were exposed to a 405 nm laser for 5 s. Subsequently, PA‐GFP activation was monitored by the appearance of the GFP signal using the 488 nm laser. Photobleaching of the activated PA‐GFP was applied to a specific nuclear area by subjecting it to 30 iterations of high‐intensity 488 nm laser exposure. Images were acquired before and after the photoactivation step and before and after the photobleaching procedure using a confocal microscope (LEICA SP8, Wetzlar, Germany).

## Results

### Designing a cellular model to study H1‐4 functions

In this study, we successfully generated a transgenic mESC model of a Rahman mutation (c.430dupG) in the linker histone H1.4. H1.4 gene and peptide sequences are evolutionarily well conserved in mammals. Human and mouse H1.4 genes display 94% identity at the amino acid level and 86% at the nucleotide level. Nevertheless, the frameshifts caused by the Rahman duplication/deletion mutations in the human H1.4 CDS do not result in the same 38 amino acids long acidic tail in mouse H1.4. To address this disparity, we designed an H1.4 targeting vector (TV) to replace the endogenous mouse H1.4 gene with its human counterpart. Our model incorporated three distinct versions of the human H1‐4 protein: the wild‐type, a variant carrying the prevalent Rahman mutation, and another variant with a truncation of 75 amino acids within the CTD (Table 3 and Fig. 1B,C). These different versions allow assessment of the impact of specific alterations on the functional properties of the H1.4 gene. Additionally, the H1.4 gene incorporated into the H1.4 TV contained a silent mutation that introduced a novel HindIII recognition sequence for subsequent ESC colony screening. This unique HindIII recognition sequence was utilized as a molecular marker to assess the zygosity of the H1.4 gene in our model. Recombination‐mediated insertion of the construct into the H1.4 locus results in an initial knockout of the mouse H1.4 allele. The presence of two polyadenylation signals within the neomycin cassette acts as a termination signal, preventing the expression of the downstream H1.4 gene. Cre‐mediated excision of the floxed neomycin gene deletes the polyadenylation sites and the neomycin gene and subsequently activates the expression of the downstream PA‐GFP‐H1.4 gene. As a result, the PA‐GFP‐H1.4 gene will be exclusively expressed under the control of the mouse endogenous promoter, enabling precise regulation of H1.4 gene expression in our model system. This conditional expression feature provides precise control over the expression and functionality of the H1.4 gene in our cellular model. Also, the linker histone H1s have been fused with GFP protein previously and were shown to be associated with chromatin and behave identically to the endogenous H1 variants [24], clearly showing that fusion of GFP to H1 does not affect the function or localization of the linker histones. Furthermore, the differentiation of these mES cells into distinct somatic cell types will shed light on the roles of both the H1.4 Rahman syndrome mutant and the CTD of H1.4 in the differentiation process.

**Table 3:** Cells and clones used and created in this study.

Genotype	Description
Control	Wild‐type and unmodified mouse B6/BLU ES cells (ATCC SCRC‐1019)
LSL	The lox‐stop‐lox (LSL) cassette includes a promoterless neomycin gene followed by BGH and SV40 polyadenylation signals flanked by loxP sites. Insertion of this cassette upstream of the coding sequence results in neomycin expression driven by the endogenous H1.4 promoter, whereas H1.4 expression is prevented. Excision of the LSL cassette by Cre‐mediated recombination enables the expression of the downstream modified H1.4 gene variants
Rahman‐Het Rahman‐Hom	mESC clones where one or both copies of the mouse H1.4 coding sequence were replaced by a construct bearing the human H1.4 CDS with the Rahman syndrome mutation (c.430dupG)
WT‐Het WT‐Hom	mESC clones where one or both copies of the mouse H1.4 coding sequence were replaced by a construct bearing the WT human H1.4 CDS
Truncated‐Het Truncated‐Hom	mESC clones where one or both copies of the mouse H1.4 coding sequence were replaced by a construct bearing the human H1.4 CDS with a premature STOP codon added at the Rahman mutation site, resulting in the expression of 144 amino acids long truncated H1.4 protein

### Genetic and phenotypic characterization of the mESC clones

The successful integration of the labeled H1.4 gene at the mouse endogenous locus was confirmed through PCR analysis of both the 3′ (Fig. 1D) and 5′ (Fig. 1E) homology arms. The PCR results indicated that the PA‐GFP‐H1.4 fusion gene has effectively replaced the mouse endogenous H1.4 gene. To further characterize the selected clones, we examined the H1.4 zygosity at the mouse locus using the novel HindIII recognition sequence present in the human H1.4 gene. Digestion analysis of the PCR products derived from the 3′ homology arm with the HindIII enzyme (Fig. 1D) allowed us to identify the H1.4 homozygous and heterozygous clones. We selected one homozygous and one heterozygous clone for each mutation for further investigation.

**Fig. 2:**
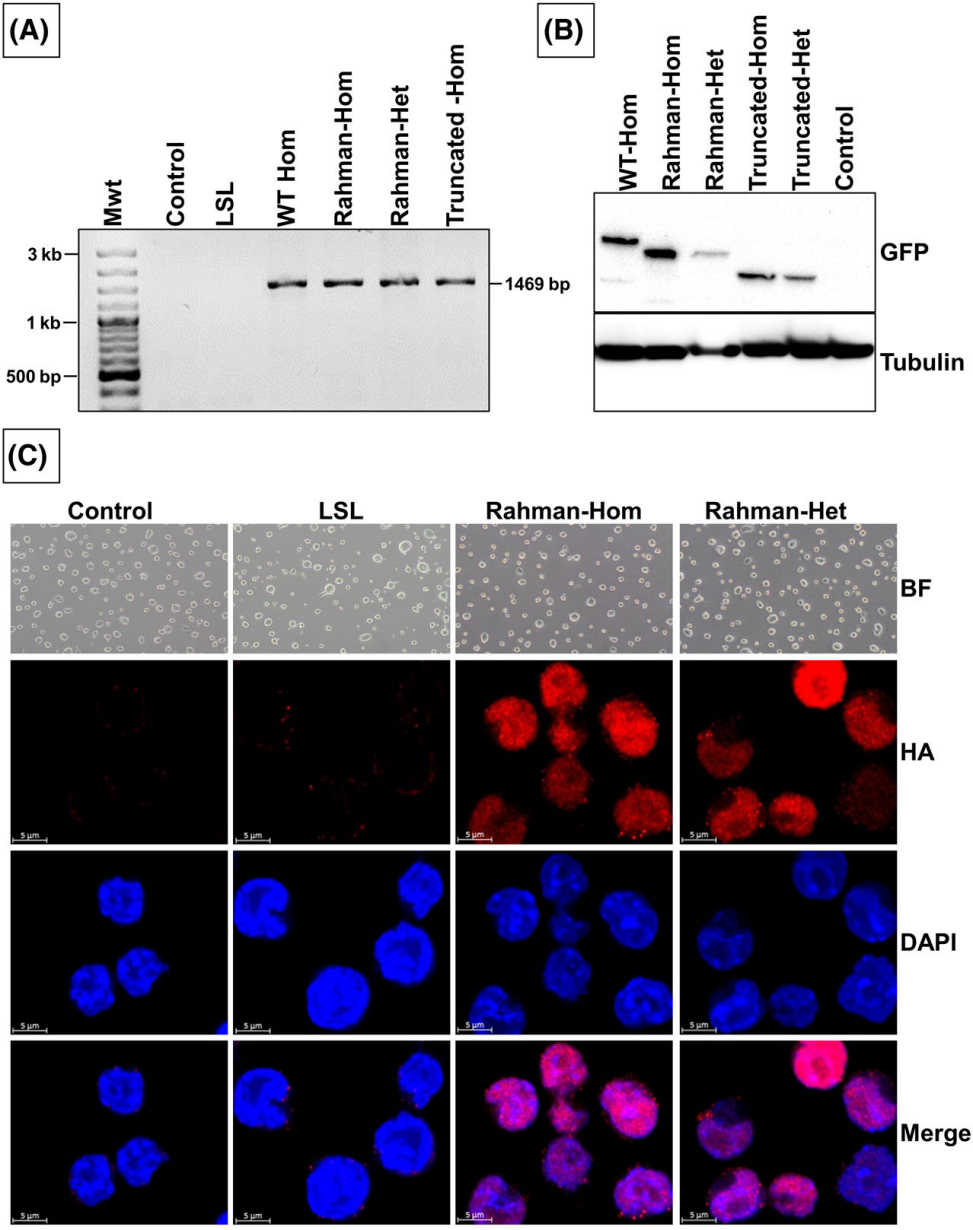
Characterization of transgenic cells. (A) PA‐GFP‐H1.4 expression analysis by using RT‐PCR: After treating the cells with adeno‐Cre, the expression of the fusion protein PA‐GFP‐H1.4 was analyzed by RT‐PCR, confirming the presence of the target gene at the mRNA level. (B) Western blot analysis of the PA‐GFP‐H1.4 fusion protein using an anti‐GFP antibody shows the expression and stability of the fusion proteins. (C) Immunofluorescence analysis of the PA‐GFP‐H1.4 protein: The upper part of the figure showcases bright field images of colonies derived from different clones. In the lower part, the immunofluorescence analysis of the PA‐GFP‐H1.4 protein is depicted using an antibody specific to the HA tag. Scale bar: 5 μm.

**Fig. 3:**
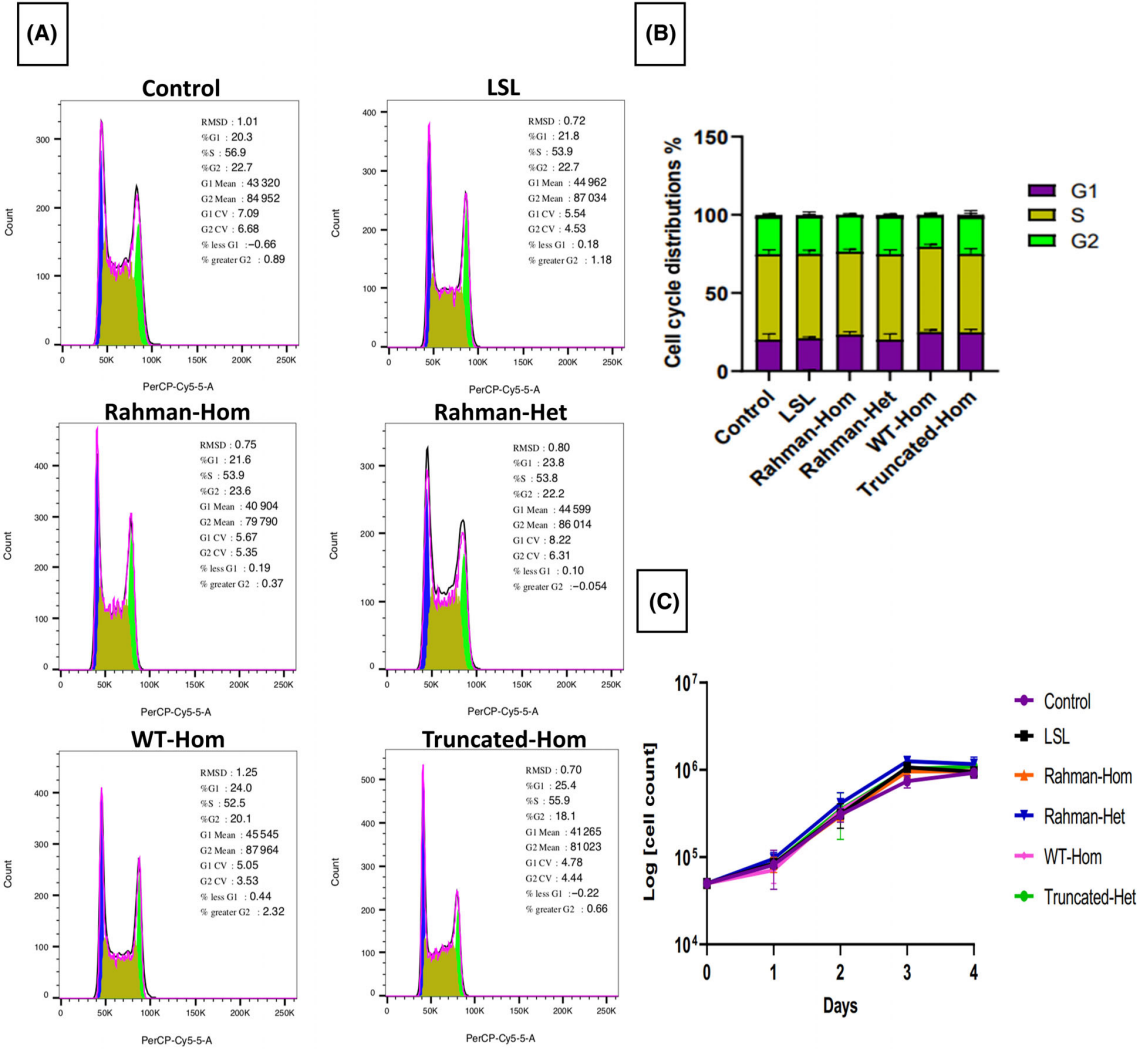
Cell cycle and growth curve analysis. (A) Individual FACS profiles from representative experiments are shown. Analysis of DNA content was performed using the Per‐CP Cy5.5 channel by flow cytometry. Cell cycle analysis was performed by flowjo v10 (FlowJo) with the Watson Pragmatic algorithm. (B) The percentage of cell cycle distribution is presented as determined by FACS. The results represent the average of three independent experiments (*n* = 3). Analysis was performed using graphpad prism, (San Diego, CA, USA) and the error bars represent SD. (C) Growth curve analysis displaying the cell count numbers of different clones over 4 days. The results represent the average of three independent experiments (*n* = 3; error bars represent SEM).

The transgenic cells generated here display the conditional expression of the transgene upon Cre recombination. The expression of the PA‐GFP‐H1‐4 fusion protein was induced by adeno‐cre‐mediated recombination. To confirm the expression of the PA‐GFP‐H1.4 gene, both RT‐PCR and western blot analyses were conducted (Fig. 2A,B). RT‐PCR data demonstrated the presence of the fusion protein in the treated clones. Moreover, the western blot analysis (Fig. 2B) provided additional evidence for the expression of the fusion protein PA‐GFP‐H1‐4, revealing distinct protein sizes due to the various truncations in the H1.4. Furthermore, RT‐PCR analysis of the mouse H1.4 gene expression demonstrated its complete loss in the homozygous clones. As expected, mouse H1.4 expression was detected in control cells and the heterozygous clones (Fig. S2A). This observation provides compelling evidence for successfully and efficiently replacing the mouse H1.4 gene with its human counterpart. Immunofluorescence utilizing the HA tag was employed to validate the proper expression and localization of the PA‐GFP‐H1‐4 protein, as depicted in Fig. 2C and Fig. S2B. The HA‐tagged PA‐GFP‐H1‐4 protein was detected inside the nucleus, confirming successful expression and localization. Moreover, cell cycle analysis (Fig. 3A,B) of these clones revealed no significant differences compared to the parental B6/BLU ES cells, indicating that the introduction of the PA‐GFP‐H1.4 gene or the presence of H1.4 mutations did not impact cell cycle progression. Furthermore, when comparing the proliferation of the generated clones with the parental cells, no significant changes were observed (Fig. 3C). This result is consistent with the cell cycle analysis, as no significant changes were observed when comparing the cell cycle analysis results between the generated clones and the parental clone. This indicates that the clones carrying the mutation are stable and exhibit typical proliferation rates.

**Fig. 4:**
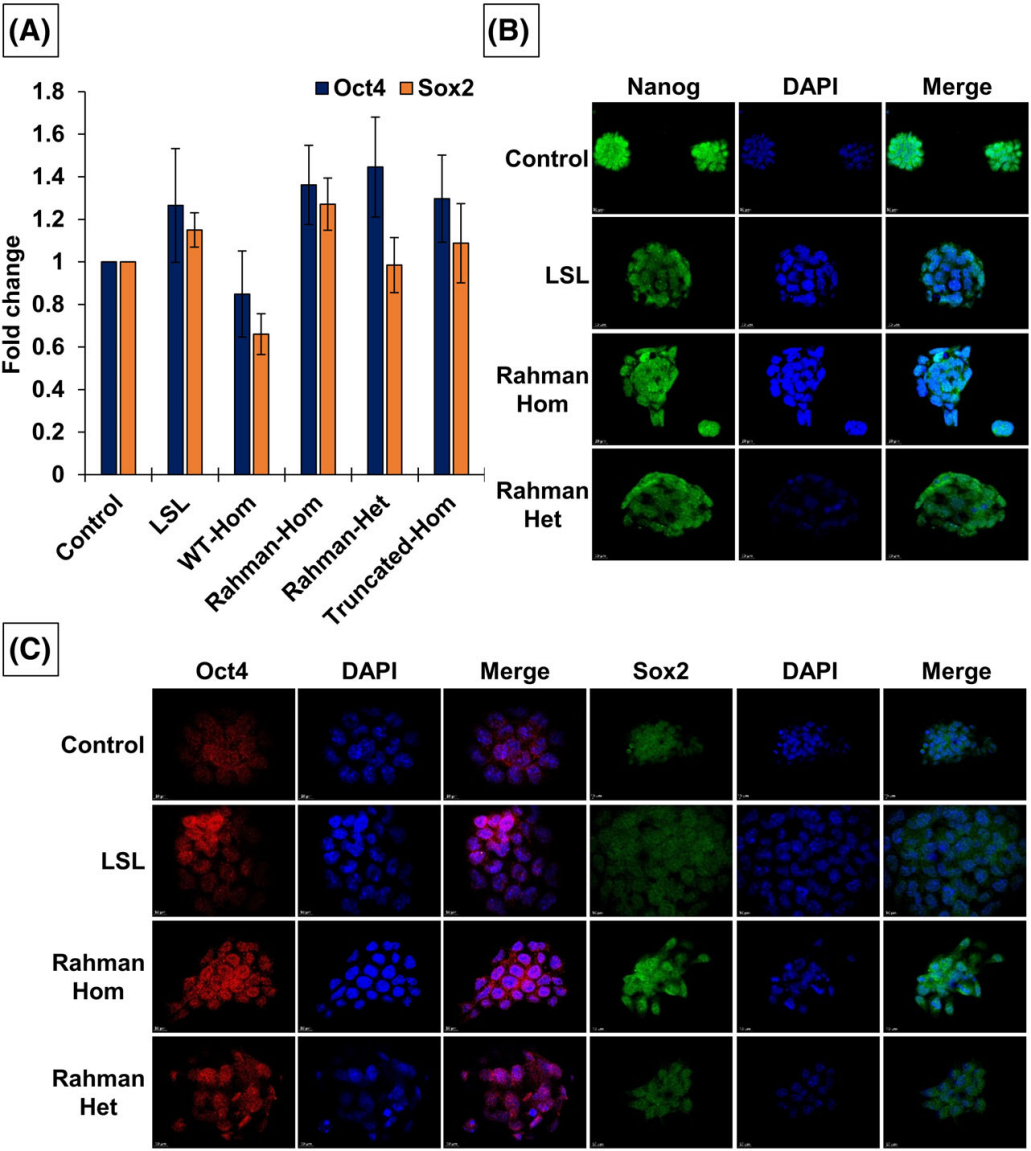
Pluripotency markers expression analysis. (A) Analysis of the pluripotency markers Sox2 and Oct4 gene expression in the generated clones using RT‐qPCR. The results represent the average of three independent experiments (*n* = 3, error bars represent SEM). (B) Immunofluorescence analysis of the pluripotency marker Nanog. Scale bar: 10 μm. (C) Immunofluorescence analysis of the pluripotency markers Oct4 (right panel) and Sox2 (left panel). Scale bar: 10 μm.

To evaluate the impact of the mutation on pluripotency marker expression, we examined the levels of pluripotency markers using quantitative qPCR and immunofluorescence techniques. Our objective was to assess the expression of these markers in the cells, particularly in the presence of the mutation. The qPCR results revealed that the cells, despite the absence of H1‐4 or in the presence of the mutated fusion protein PA‐GFP‐H1‐4, exhibited robust expression of Oct4 and Sox2, indicating the maintenance of pluripotency gene expression (Fig. 4A). These findings suggest that the cells retained their embryonic characteristics and remained undifferentiated, capable of self‐renewal and differentiation into various cell lineages. Immunofluorescence analysis further confirmed the expression of pluripotency markers in the cells carrying the mutations (Fig. 4B,C and Fig. S2C,D). Despite the mutation, the cells maintained their embryonic properties, suggesting their continued utility as a valuable model for investigating developmental processes and exploring lineage‐specific differentiations.

### Fibroblast differentiation

**Fig. 5:**
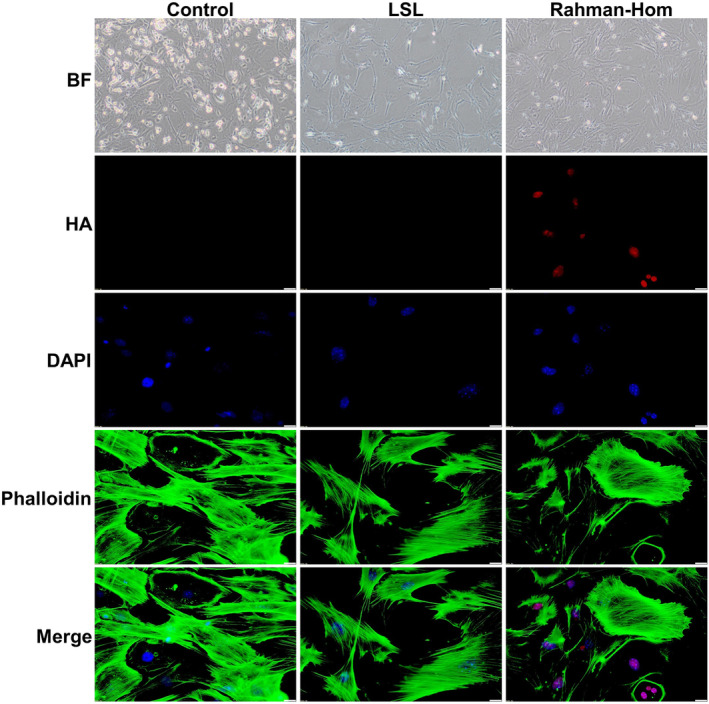
Differentiation of transgenic clones into fibroblasts. Mouse embryonic stem cells from the generated clones were cultured at low density with retinoic acid for 10 days, forming a compact monolayer, then trypsinized and reseeded to allow fibroblast attachment, and propagated further in DMEM with 10% FBS. The top panel shows bright‐field images of differentiated fibroblasts from different clones. In the lower part, the staining of cells with the anti‐HA antibody and Alexa Fluor™ 488 phalloidin (for F‐actin staining) are depicted. Scale bar: 20 μm.

Working with ES cells offers a significant advantage due to their remarkable potential to differentiate into various cell types. Fibroblasts derived from Rahman syndrome patients displayed different epigenetic profiles when compared to the control samples [19]. In our study, we aimed to assess the differentiation capacity of our model mES cells into fibroblasts‐like cells. Our results demonstrated the successful differentiation of our model ES cells into fibroblast‐like cells, validated through immunofluorescence analysis of F‐actin. The resultant fibroblast‐like cells have a characteristic spindle shape and contain actin stress fibers that stain positively for phalloidin (Fig. 5). This feature has been utilized to characterize mouse embryonic fibroblast (MEF) cells [25, 26]. In addition, cells derived from the Rahman homozygous clone showed proper expression of the PA‐GFP‐H1.4 fusion protein, as demonstrated by the immunofluorescence against the HA tag. These findings conclusively establish that our model mES clones effectively converted into a fibroblast‐like phenotype. This outcome not only confirms the cells' potential for directed differentiation but also underscores their suitability for in‐depth research and further investigations.

## Discussion

The cellular models developed here mainly concern Rahman syndrome, a rare genetic disorder resulting from *de novo* occurring mutations on one allele of the human H1.4 gene coding sequence. Nevertheless, the implications of these cellular models will eventually extend our humble knowledge about the functions of H1‐4. Additionally, the cell lines where we deleted a substantial portion of the H1.4 CTD will allow us to study the specific functions of this domain. The mutations that cause Rahman syndrome localize to the part of the H1.4 gene coding for CTD, resulting in a reduction in both size and net charge, specifically reducing the wild‐type CTD's net charge from +41 to approximately +4 [27]. On the other hand, a simple truncation of the CTD, starting from the region where Rahman mutations occur, leads to a decrease in size and has a net charge of +10 (Fig. 1B,C) without generating the specific 38 amino acid stretch found in Rahman patients. Therefore, our model incorporated this H1.4 truncation mutation to distinguish whether the observed effects result primarily from the shorter CTD or occur due to the characteristic 38 amino acid stretch at the end of the CTD. Both homozygous and heterozygous cell lines were generated to comprehensively investigate these effects, creating a robust model for detailed analysis. Homozygous clones will allow us to understand whether the dosage of the Rahman mutant can amplify the effects. Furthermore, the cell line carrying the wild‐type human H1.4 serves as a control for evaluating the replacement of the mouse gene with the labeled human counterpart.

Evaluating cell viability in the presence of the homozygous mutated H1.4 gene provides significant insights, indicating that the elevated dosage of the Rahman mutant and truncated H1‐4 proteins does not affect the viability of mES cells. The described model is highly prized because the absence of H1‐4 or expression of the mutated protein fused with PA‐GFP has no effects on the pluripotency of stem cells or their proliferation rate and differentiation potential. The photoactivation of PA‐GFP within cells expressing the PA‐GFP‐H1.4 fusion protein revealed its specific localization within the nucleus and its ability to undergo photoactivation upon exposure to a 405 nm laser. Moreover, following photobleaching of the photoactivated PA‐GFP‐H1.4 protein, the GFP signal was fully recovered, confirming that the fusion protein is trackable (Fig. S3). However, it is essential to perform a robust FRAP (fluorescence recovery after photobleaching) assay to accurately quantify the exchange rate of labeled H1.4 and its residence time. It is also worth mentioning here that the expression of the H1.4 gene in stem cells is low compared to differentiated cells [28], and stem cells exhibit the lowest H1 to nucleosome ratio, indicating a potentially reduced functionality of the H1‐4 linker histone in stem cells [29, 30]. This observation could explain the stability of our model in mES cells. Since the model was developed on mouse ESCs, it can generate different cell types using appropriate differentiation protocols. In this article, we demonstrated the ability of our model to differentiate into fibroblasts. However, future experiments should focus on neuronal differentiation. This is supported by initial data from rat hippocampal neurons transfected with the mutated H1.4, which showed a unique subnuclear distribution and enlarged nuclei [31]. The neuronal firing rate was also reduced compared to neurons overexpressing the wild‐type human H1‐4 protein [31].

Methylation analysis on leukocytes expressing the mutant form of H1‐4 revealed a high enrichment of genes associated with neurological, immunological, and cell adhesion/membrane function pathways compared to the control [31]. This is consistent with the clinical and phenotypic features of the syndrome. Moreover, methylome analysis in affected individuals showed a specific epi‐signature characterized by hypomethylation profiles in gene regions predominantly expressed in the brain [19, 32]. These results suggest that the truncated H1‐4 protein modulates the epigenome and affects the genes required for proper neuronal development [27]. We believe our Rahman model could be used in many aspects to study the biology of H1‐4. Since the model was created in mES cells, they can be differentiated into various cell types, such as neurons or fibroblast cells. This provides the opportunity to perform RNA sequencing analysis in both differentiated and undifferentiated cell states, allowing for investigating the effects of the H1.4 mutation on gene expression. In the current model, H1‐4 was labeled with PA‐GFP, enabling the real‐time monitoring of chromatin dynamics using techniques like FRAP or FLIP (fluorescence loss in photobleaching). Moreover, GFP and HA‐tag pulldown can be employed to explore the physical interactions between the mutant H1‐4 protein and other proteins. Furthermore, these transgenic mES cells can be utilized to establish a mouse model, enabling in‐depth analysis of the mutation's presence in various cells and tissues.

## Conflict of interest

The authors declare no conflict of interest.

### Peer review

The peer review history for this article is available at https://www.webofscience.com/api/gateway/wos/peer‐review/10.1002/2211‐5463.13750.

## Author contributions

SD, INL, and MKD conceived and supervised the study; AAAA and INL designed the experiments; AAAA, EOS, MEE, and TB performed the experiments; INL and AAAA wrote the manuscript; SD, MKD, DA, AH, HA, and TY made manuscript revisions; and EK helped with the FRAP experiment. SD, DA, and INL acquired the funding.

## Supporting information


**Fig. S1.** Amino acid sequence of PA‐GFP‐H1.4
**Fig. S2.** Characterization of cells expressing mutant H1.4.
**Fig. S3.** Fluorescence recovery after photobleaching (FRAP).

## Data Availability

The data that support the findings of this study are available from the corresponding author (imtiaznisar.lone@ibg.edu.tr, imtiaznisar.lone@gmail.com) upon reasonable request.
