# Supplementary material for: Development of a mouse embryonic stem cell model for investigating the functions of the linker histone H1‐4
Source: FEBS Open Bio. 2024 Jan 11;14(2):309–21. doi: 10.1002/2211-5463.13750 (PMC10839353; doi:10.1002/2211-5463.13750)
Supplement: Supplementary file 1 — Fig. S1. Amino acid sequence of PA‐GFP‐H1.4 Fig. S2. Characterization of cells expressing mutant H1.4. Fig. S3. Fluorescence recovery after photobleaching (FRAP). [file FEB4-14-309-s001.docx]

**
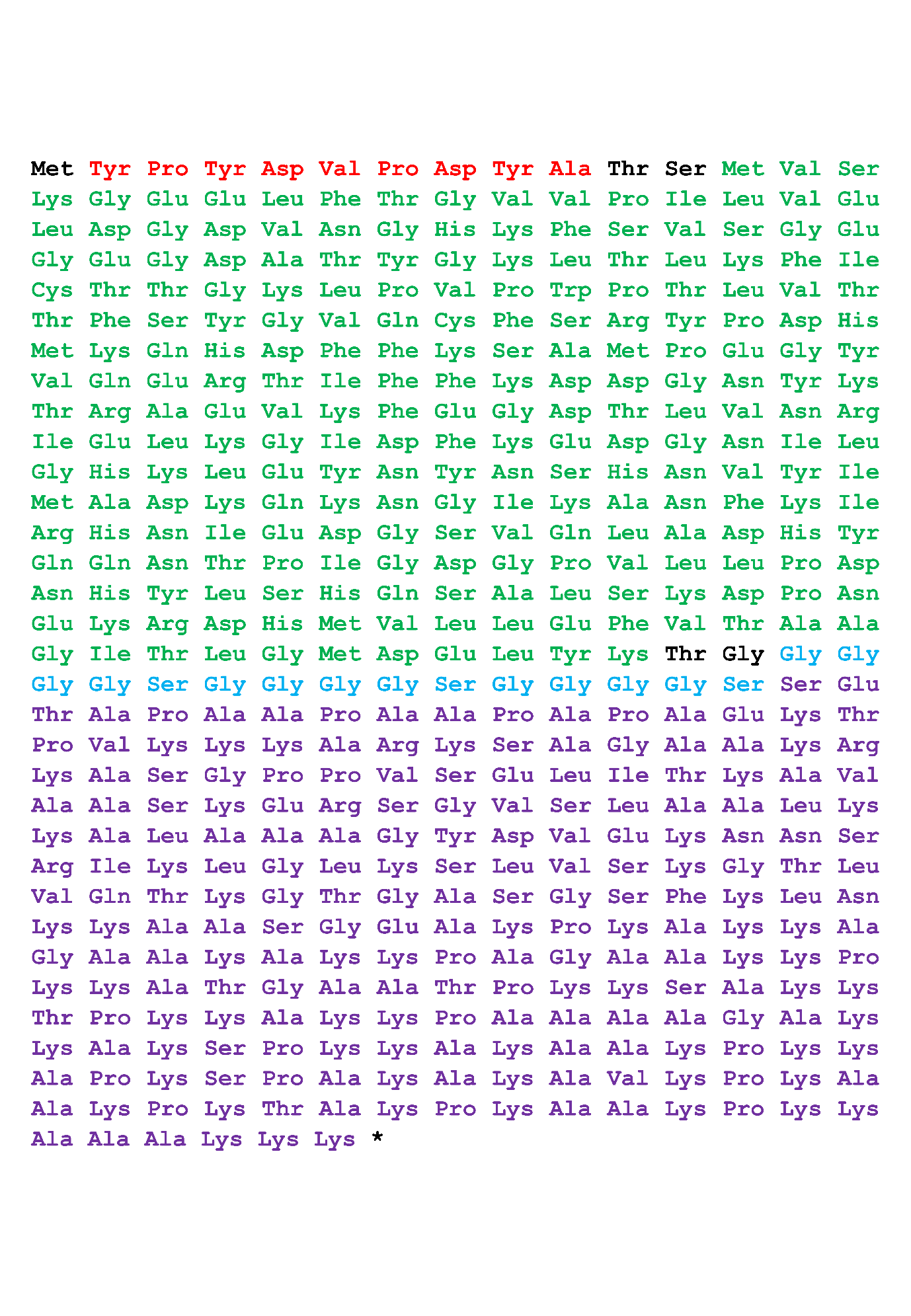
**

**Supplementary Figure 1: Amino Acid Sequence of PA-GFP-H1.4**

The full amino acid sequence of the PA-GFP-H1.4 protein is color-coded, with red representing the HA tag amino acid sequence, green for the PA-GFP amino acid sequence, blue for the GC linker amino acid sequence, and purple for the wild-type H1.4 amino acid sequence. The initiator amino acid is Methionine (Met), and the SpeI recognition sequence is found at Thr Ser, while the AgeI recognition sequence is located at Thr Gly all in black color. A stop codon (*) marks the end of the protein sequence.


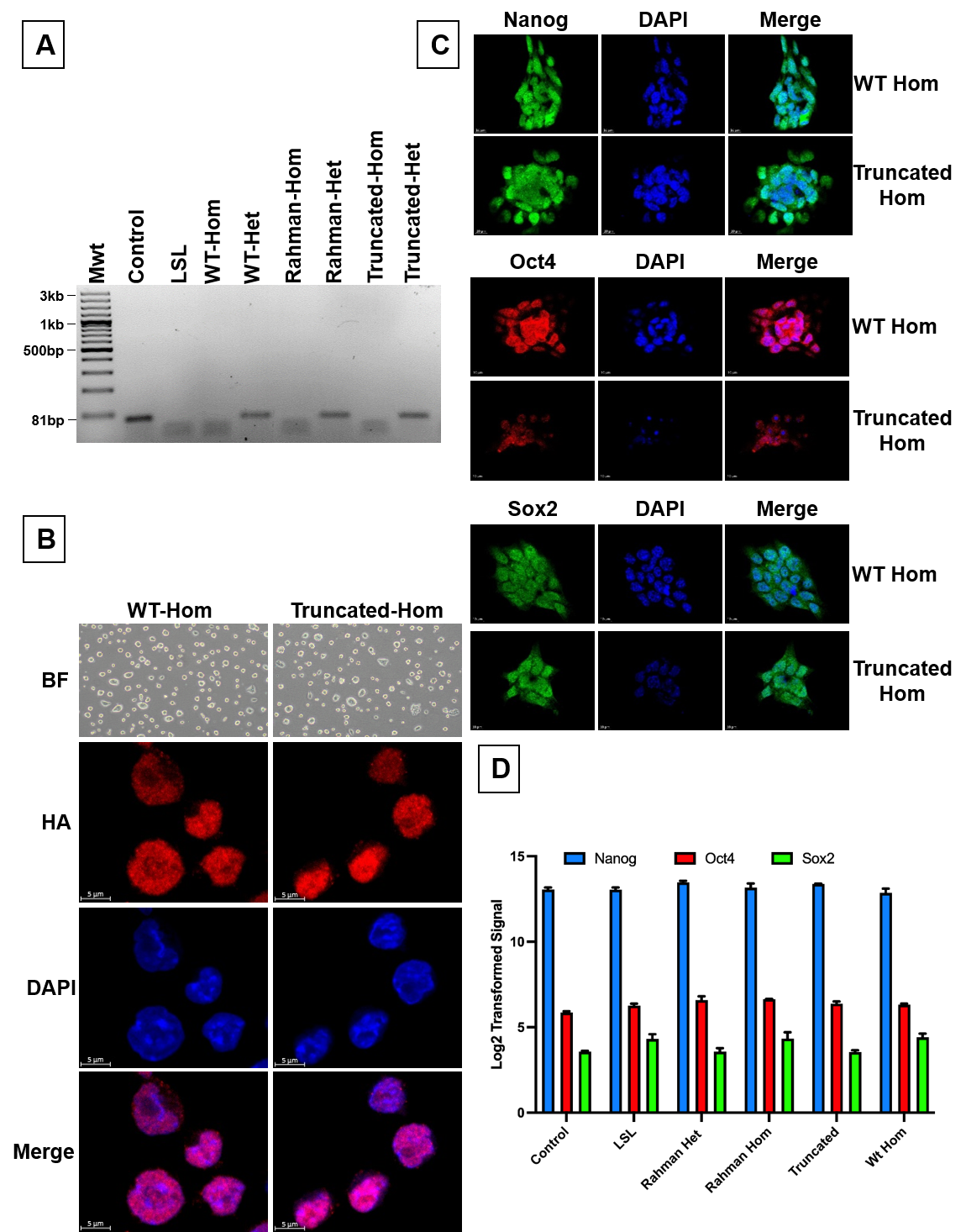


**Supplementary Figure 2: Characterization of Cells expressing mutant H1.4**

**A:** Analysis of H1.4 gene expression in the generated clones using RT-PCR. All heterozygous clones express one copy of the endogenous mouse H1.4. **B:** The upper part of the figure showcases bright field images of colonies derived from different clones. In the lower part, the immunofluorescence analysis of the PA-GFP-H1.4 protein is depicted using an antibody specific to the HA tag. Scale bar: 5µm. **C:** Expression of the pluripotency markers Oct4 (Top left panel), Sox2 (Top right panel), and Nanog (bottom panel) was performed by immunofluorescence. All three markers showed the expression as expected for a pluripotent cell. Scale bar: 10µm. **D:** Log2 transformed signal of pluripotency markers depicted in Figure 4B and 4C; and supplementary Figure 2C were quantified using ImageJ software. The data represents two independent experiments (n = 2). On average 200 cells from each clone were analyzed for each marker.

**
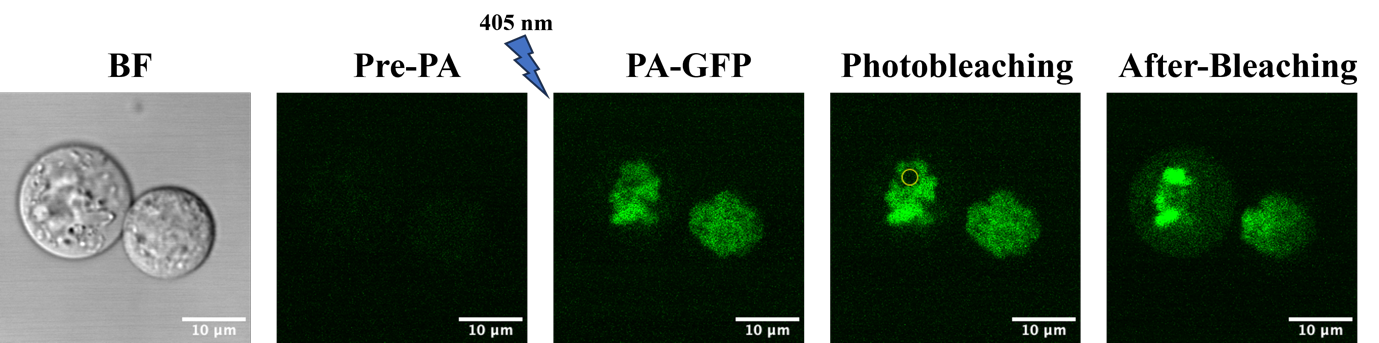
**

**Supplementary Figure 3: Fluorescence Recovery After Photobleaching (FRAP):**

Cells expressing PA-GFP-H1.4 were imaged before the activation of PA-GFP (Bright field and Pre-PA), after the activation (PA-GFP), during photobleaching, and during recovery after photobleaching. The photobleached area is indicated by a dotted circle. Scale Bar: 10µm.
